# Gas-liquid partitioning and olfactory interplay between isoamyl alcohol and typical odorants in binary hydroalcoholic models of navel orange brandy

**DOI:** 10.3389/fnut.2026.1927483

**Published:** 2026-08-13

**Authors:** Zhenzhen Huang, Zhuoyue Wu, Shasha Qu, Wenzhao Liu, Yingjie Feng, Jingnan Ren, Jinchu Yang, Yongming Xu, Gang Fan

**Affiliations:** 1 Technology Center, China Tobacco Henan Industrial Co., Ltd., Zhengzhou, Henan, China; 2 Key Laboratory of Environment Correlative Dietology, Ministry of Education, College of Food Science and Technology, Huazhong Agricultural University, Wuhan, Hubei, China

**Keywords:** binary hydroalcoholic model, fusel oil, gas-liquid partitioning, isoamyl alcohol, navel orange brandy, olfactory interplay, typical odorants

## Abstract

Intermolecular interactions among volatile typical odorants define the characteristic sensory profile of navel orange brandy, with isoamyl alcohol-the dominant fusel higher alcohol-functioning as a pivotal flavor regulator. Excess isoamyl alcohol blunts distinct citrus fruity notes and destroys organoleptic harmony; nevertheless, systematic mechanistic explorations targeting citrus brandy remain insufficient. Herein, simplified 54% (v/v) binary hydroalcoholic models were established to exclude complicated coexisting matrix interference, allowing exclusive dissection of pairwise odorant interactions. A dual analytical workflow combining HS-SPME-GC-MS quantification and sigmoidal olfactory threshold fitting was constructed to elucidate concentration-dependent (0–800 mg/L isoamyl alcohol) variations in gas-liquid partitioning and olfactory interplay across 18 citrus typical odorants. In binary hydroalcoholic systems, isoamyl alcohol continuously hampered the volatilization of fatty acid ethyl esters, and inhibitory potency declined progressively as carbon chains elongated. This fusel alcohol imposed dual concentration-mediated effects on linear aliphatic alcohols: concentrations ≤ 400 mg/L boosted odorant release into the headspace, while higher dosages suppressed partitioning, and such bidirectional shifts were more prominent for short-chain analogs. Acyclic terpenols suffered steady volatility attenuation with elevated isoamyl alcohol, whereas monocyclic *D*-limonene and α-terpineol underwent initial promotion followed by inhibition, with respective turning points at 400 mg/L and 600 mg/L. Polar minor volatiles (furfural, 4-ethylphenol, 3-hydroxy-2-butanone) exhibited gradual depletion in headspace concentration, while weakly polar styrene displayed consistent partitioning elevation. Olfactory evaluation of all 18 binary blends identified seven masking, seven additive and four synergistic binary pairs. Overall, isoamyl alcohol concealed ester-originated fruity aromas yet intensified the sensory perception of alcoholic and cyclic terpene notes. The quantitative datasets acquired in this research deliver reliable theoretical basis and technical guidance for precise isoamyl alcohol modulation in citrus brandy manufacturing. Notably, the present work only addresses pairwise interactions within simplified binary hydroalcoholic models; multi-component flavor coupling effects and verification on naturally aged authentic brandy will be explored in subsequent research.

## 1 Introduction

Navel orange brandy is a distinctive citrus fruit distilled spirit with unique market advantages, featuring an elegant taste and fresh natural citrus aroma. It is primarily produced via fermentation and distillation using fresh navel oranges as the main raw material (1). Terpenoids, which are abundant in navel orange fruits, exert a decisive effect on shaping the characteristic citrus aroma of navel orange brandy (2). Furthermore, esters, aliphatic alcohols, aldehydes and ketones present in this distilled liquor are also essential volatile components closely linked to consumer acceptance and commercial grading (1, 3, 4). The concentration and intermolecular interactions of these volatile substances further define the overall flavor characteristics and market competitiveness of the final product (5).

As an important fermentation-derived higher alcohol, isoamyl alcohol is a common flavor ingredient in navel orange brandy (6, 7). Appropriate content of isoamyl alcohol can enrich flavor layers and improve the taste structure of distilled spirits (8). Nevertheless, restricted by raw material traits and fermentation techniques, isoamyl alcohol tends to accumulate excessively in practical production (9). Excess isoamyl alcohol will break the original balanced aroma system, cover up fresh citrus aroma, and bring adverse physical discomfort after drinking, which has become a key obstacle limiting quality improvement and industrial development of citrus distilled spirits (10, 11).

As mentioned above, existing studies concerning isoamyl alcohol in navel orange brandy mainly focus on its content analysis (12), aroma properties (13), olfactory threshold (14), biosynthesis pathways (15) and basic sensory effects (16). These findings provide fundamental theoretical support for clarifying the flavor functions of isoamyl alcohol and optimizing processing techniques to enhance the overall quality of navel orange brandy. However, studies regarding the interactive and regulatory effects of isoamyl alcohol on the core characteristic flavor substances of navel orange brandy remain limited. This research gap hinders the development of targeted flavor regulation strategies for industrial production.

Notably, numerous studies have explored the properties of isoamyl alcohol in Chinese grain baijiu and grape wine (17–19). Excess isoamyl alcohol modulates matrix polarity and intermolecular interactions, lowering gas–liquid partition coefficients of ester aroma compounds and thereby suppressing their release into the headspace (20). In terms of its interaction with aliphatic alcohols, isoamyl alcohol presents concentration-dependent regulatory characteristics within wine matrices. Moderate contents help enrich aroma layers and coordinate overall flavor, while excessive amounts break the balance of alcoholic flavor substances, weaken favorable aliphatic alcohol-related aromas and reduce the comprehensive sensory quality of finished products (21). However, traditional wines differ markedly from navel orange brandy in chemical composition and core aroma components (22). Consequently, existing research conclusions cannot be directly applied to citrus distilled spirits.

To clarify the influence of isoamyl alcohol on the flavor quality of navel orange brandy, this study constructed standardized 54% (v/v) binary hydroalcoholic simulation systems. Based on previous odor activity value calculation and aroma recombination experiments (23), eighteen characteristic aroma compounds in navel orange brandy were selected as target analytes, mainly covering terpenoids and other dominant volatile flavor constituents. HS-SPME-GC-MS was employed to characterize their headspace distribution at five isoamyl alcohol concentration gradients, aiming to clarify the changing rules of these aroma components. Combined with sigmoidal curve fitting and 3-AFC sensory evaluation, three types of olfactory interaction modes were classified to further analyze flavor interaction relationships. This dual-dimensional quantitative system provides reliable data support and practical technical guidance for targeted flavor regulation and quality improvement of navel orange brandy.

## 2 Materials and methods

### 2.1 Chemicals

Isoamyl alcohol, isobutanol, propanol, butanol, hexanol, ethyl acetate, ethyl isovalerate, ethyl hexanoate, ethyl octanoate, ethyl nonanoate, ethyl laurate, *D*-limonene, linalool, geraniol, α-terpineol, octanol, decanol, furfural, 3-hydroxy-2-butanone, styrene and 4-ethylphenol reference standards were purchased from Sigma-Aldrich (Shanghai, China). Analytical-grade absolute ethanol and sodium chloride were supplied by Sinopharm Chemical Reagent Co., Ltd.

### 2.2 Sample preparation

A 54% (v/v) ethanol-water solution served as the uniform simulation matrix, consistent with the alcohol strength of mainstream commercial navel orange brandy. Separate binary mixtures containing a single fixed odorant and gradient isoamyl alcohol concentrations were prepared to avoid multi-component coupling interference. Target odorants and their concentrations were screened and calibrated based on odor activity value (OAV) calculation, aroma recombination and omission tests according to our previous research (23). Final concentrations were normalized based on GC-MS peak areas (Table 1). Five isoamyl alcohol gradients (0, 200, 400, 600, 800 mg/L) were set referring to the reported fusel alcohol levels in fruit and grain spirits (24). All stock standard solutions were stored under constant temperature and dark conditions prior to analysis (25).

**TABLE 1:** Concentrations of tested typical odorants in binary hydroalcoholic models.

No.	Typical odorants	Concentration (μg/L)
1	Ethyl acetate	8974900.00
2	Ethyl isovalerate	1719.36
3	Ethyl hexanoate	57776.33
4	Ethyl octanoate	862655.00
5	Ethyl nonanoate	957.41
6	Ethyl laurate	2790.37
7	Butanol	13432.50
8	Hexanol	1627.82
9	Octanol	1645.73
10	Decanol	1237.28
11	Linalool	768.55
12	Geraniol	3375.60
13	*D*-Limonene	8018.00
14	α-Terpineol	17670.00
15	Furfural	5771.00
16	Styrene	2991.63
17	4-Ethylphenol	1005.95
18	3-Hydroxy-2-butanone	1000.00

### 2.3 HS-SPME-GC-MS analysis

A 50/30 μm DVB/CAR/PDMS composite extraction fiber was used for headspace sampling. Ten milliliters of sample and 1 g NaCl were transferred into a 40 mL headspace vial, equilibrated at 45 °C for 5 min, followed by 40 min of stirring adsorption and 5 min of thermal desorption in the GC inlet. Each treatment was prepared in three independent parallel headspace vials for individual extraction and analysis.

An Agilent 6890N gas chromatograph coupled with a 5975B mass spectrometer equipped with a DA-WAX polar capillary column was used for compound separation. Helium was used as the carrier gas at a constant flow rate of 1.2 mL/min under splitless injection mode; inlet temperature was set to 250 °C. The oven temperature program: held at 40 °C for 2 min, then ramp to 200 °C at 8 °C/min. Ion source temperature was 230 °C, transfer line temperature 150 °C, electron ionization energy 70 eV, and mass scanning range m/z 35–450.

Preliminary single-component tests confirmed that all tested concentrations of target odorants were within the suitable adsorption range of the extraction fiber, and fiber saturation could be effectively avoided under the established extraction conditions. Comparative analysis of single-component and mixed systems further indicated that the influence of competitive adsorption among analytes was negligible at the adopted experimental concentrations. Repeatability tests were performed to ensure stable detection signals and valid intergroup comparison of relative volatile release, without conducting absolute concentration quantification. The group with 0 mg/L isoamyl alcohol was defined as the blank control. The relative peak area of each treatment group compared with the blank was used to characterize the relative volatile release level of aroma compounds, and this index cannot be regarded as actual gas-liquid partition coefficient.

### 2.4 Olfactory threshold quantification

The three-alternative forced-choice (3-AFC) sensory method was adopted to measure the olfactory thresholds of single odorants and binary mixtures in 54% (v/v) hydroalcoholic models, following the testing protocol reported by Chen et al. (26). The binary mixture threshold is defined as the minimum detectable concentration of target odorants in mixed aroma systems. Ten trained panelists from Huazhong Agricultural University were enrolled in this sensory test, including seven females and three males with a mean age of 24 years. Prior to participation, all panelists signed written informed consent after receiving a complete briefing on the experimental protocols and potential risks associated with exposure to ethanol and volatile aroma compounds. They had received three months of systematic sensory training, and possessed stable odor discrimination ability and consistent judgment performance, which ensured the reliability of olfactory threshold evaluation results. The characteristic aroma of each typical odorant was introduced, and corresponding standard stock solutions were provided for odor familiarization. For olfactory threshold measurement of single odorants and binary mixtures, the initial concentrations of all samples are shown in Table 1, with isoamyl alcohol set at 400 mg/L. Single odorants were individually prepared into 10 two-fold dilution gradients, while binary mixtures were mixed at fixed ratios using the same initial concentrations and then subjected to 10 successive two-fold dilutions. All sensory evaluations were carried out in constantly ventilated sensory booths to reduce inhalation hazards posed by ethanol and volatile aroma compounds. The evaluations started from the highest concentration. Each test group consisted of three vials: one sample fortified with the target odorant and two blank hydroalcoholic controls. If panelists correctly identified the odor-spiked sample, testing continued with the subsequent lower concentration until consistent correct identification was no longer possible. All sensory assessments were completed over three independent replicate sessions. The raw detection probability *P* derived from panel responses was corrected using the following formula:


$$P=\frac{3\,p-1}{2}$$

where *P* = corrected detection probability, and *p* = experimentally measured raw detection probability. Calibration plots were generated with log-transformed odorant concentrations on the x-axis and corrected probability *P* on the y-axis. The obtained concentration-probability datasets were fitted to the standard sigmoidal logistic equation:


$$P=\frac{1}{1+e^{-\frac{x-C}{D}}}$$

where *P* = corrected detection probability, *x* = log-transformed analyte concentration, *C* = threshold parameter, *D* = slope parameter. All curve fittings were performed using the Levenberg–Marquardt iterative algorithm. Initial estimates of parameters *C* and *D* were assigned based on pre-determined concentration ranges and preliminary sensory test results. All fitted parameters were constrained within scientifically and sensorially reasonable bounds to avoid physically meaningless solutions. Binomial sensory response data were weighted by valid panelist sample sizes to improve fitting reliability. The goodness-of-fit was assessed using the coefficient of determination *R*^2^, which provides an overall measure of model fit for the sigmoidal curves, while its inherent limitations should be recognized when applied to binomial detection data. The olfactory threshold of each analyte was defined as the concentration value corresponding to *P* = 0.5 on the fitted curve.

The theoretical detection probability of binary mixtures was calculated via the Feller additive formula, which is widely used for predicting sensory responses of aroma mixtures in relevant studies (27):


P⁢(AB)=P⁢(A)+P⁢(B)-P⁢(A)×P⁢(B)

where *P*(A) and *P*(B) represent the corrected detection probabilities of single odorants extracted from their respective fitted sigmoidal curves, and *P*(AB) represents the theoretical detection probability of binary mixture. The Feller additive formula is established on the premise of independent olfactory detection events. It is extensively adopted to calculate theoretical binary thresholds, since it serves as a standard additive baseline to quantify genuine perceptual interactions between aroma components. Log-transformed odorant concentrations and corrected detection probability were fitted to the sigmoidal logistic equation as above. Subsequently, theoretical binary olfactory thresholds were determined from the fitted concentration-probability relationship using the calculated theoretical detection probabilities.

### 2.5 Sigmoidal curve modeling for olfactory interplay classification

Olfactory interaction modes were classified using the ratio of experimentally determined thresholds to theoretical thresholds derived from the Feller model. This classification approach has been widely recognized and applied in aroma interaction studies (28). A lower experimental threshold indicates that the odor mixture is detectable at a lower concentration, indicative of synergistic interaction. In comparison, a higher experimental threshold reflects mutual inhibition between odor molecules, namely the masking effect. In line with conventional research criteria, the detailed classification standards are defined as follows:

Ratio < 0.5: Synergistic effect;

0.5 ≤ Ratio ≤ 1: Additive effect;

Ratio > 1: Masking effect;

Ratio = 1: Neutral effect.

### 2.6 Statistical analysis

Statistical differences among groups were analyzed by one-way analysis of variance (ANOVA), followed by Duncan’s multiple range test for *post-hoc* comparison at a significance level of *p* < 0.05. Sigmoidal fitting for olfactory threshold curves and all graphical plotting were completed using professional statistical and plotting tools. Correlation coefficients of all fitted models were calculated to evaluate fitting quality.

## 3 Results

### 3.1 Concentration-dependent partitioning modulation of odorants by isoamyl alcohol

All regulatory trends presented in this study were obtained from simplified binary hydroalcoholic models (Table 1). Isoamyl alcohol imposed structure-selective and dose-dependent effects on the gas-liquid partitioning of all tested odorants, and evident differences were observed among chemical classes (Table 2). Notably, negative values in Table 2 indicate volatility promotion, while positive values refer to volatility inhibition.

**TABLE 2:** Inhibition rate of typical odorant volatilization induced by isoamyl alcohol.

No.	Category	Compound	Inhibition rate (%) (200 mg/L)	Inhibition rate (%) (400 mg/L)	Inhibition rate (%) (600 mg/L)	Inhibition rate (%) (800 mg/L)
1	Esters	Ethyl acetate	33.95 ^a^	38.67 ^a^	41.96 ^a^	42.22 ^b^
2		Ethyl isovalerate	31.74 ^a^	33.56 ^b^	40.58 ^b^	50.14 ^a^
3		Ethyl hexanoate	7.82 ^bc^	13.38 ^c^	17.16 ^e^	19.39 ^c^
4		Ethyl octanoate	5.40 ^c^	11.42 ^c^	18.64 ^d^	20.48 ^c^
5		Ethyl nonanoate	1.30 ^d^	1.72 ^d^	2.12 ^g^	3.49 ^d^
6		Ethyl laurate	0.42 ^d^	1.50 ^d^	3.80 ^f^	4.15 ^d^
1	Alcohols	Butanol	−13.57 ^a^	−10.75 ^a^	2.14 ^ab^	8.32 ^b^
2		Hexanol	−12.14 ^a^	−7.53 ^a^	14.16 ^a^	57.08 ^a^
3		Octanol	−4.71 ^a^	−18.20 ^a^	−7.03 ^b^	9.69 ^b^
4		Decanol	−2.88 ^a^	−7.71 ^a^	−3.60 ^ab^	12.19 ^b^
1	Terpenoids	Geraniol	2.16 ^c^	6.32 ^b^	10.74 ^c^	11.04 ^c^
2		Linalool	11.59 ^a^	29.48 ^a^	59.09 ^a^	63.14 ^a^
3		*D*-Limonene	−20.74 ^e^	−24.78 ^d^	25.10 ^b^	17.36 ^b^
4		α-Terpineol	−5.53 ^d^	−6.77 ^c^	−10.12 ^d^	7.68 ^d^
1	Other compounds	4-Ethylphenol	7.66	13.48	20.67	20.78
2		Styrene	−0.54	−3.06	−12.84	−26.91
3		Furfural	4.78	6.96	9.90	13.51
4		3-Hydroxy-2-butanone	2.16	6.32	15.63	20.24

Different lowercase letters denote significant differences via Duncan’s multiple range test (*p* < 0.05). Negative values denote volatility promotion by isoamyl alcohol.

#### 3.1.1 Fatty acid ethyl esters

**FIGURE 1:**
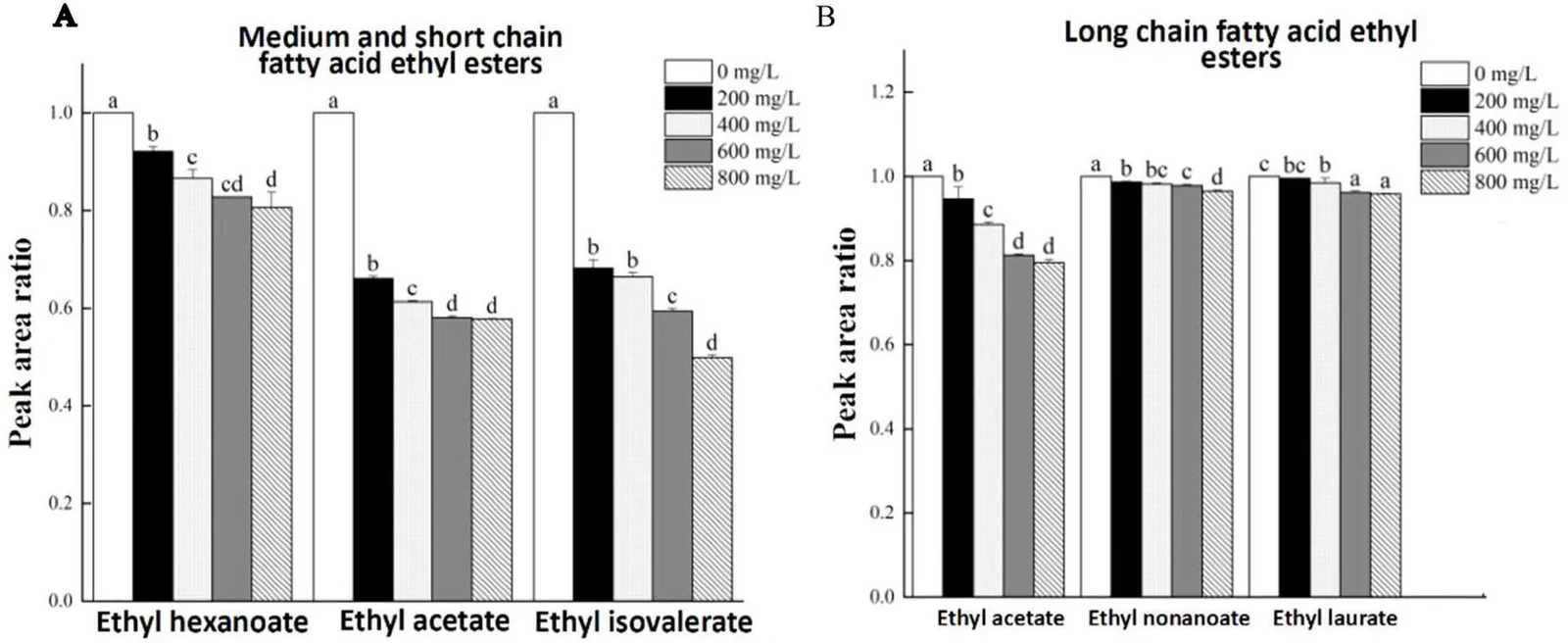
Effects of isoamyl alcohol on the volatility of fatty acid ethyl esters. **(A)** Medium- and short-chain fatty acid ethyl esters. **(B)** Long-chain fatty acid ethyl esters. Different lowercase letters within the same compound group indicate significant differences determined by Duncan’s multiple range test (*p* < 0.05).

Under binary hydroalcoholic models, isoamyl alcohol continuously inhibited the volatilization of esters, and this inhibitory effect gradually weakened with the increase of alkyl carbon chains length (Figure 1). All tested esters exhibited significant reductions in volatility (*p* < 0.05). Ethyl acetate was the most strongly inhibited, with an inhibition rate of 33.95% at an isoamyl alcohol level of 200 mg/L, while ethyl laurate achieved only 0.42% inhibition under identical conditions (Table 2).

#### 3.1.2 Linear aliphatic alcohols

**FIGURE 2:**
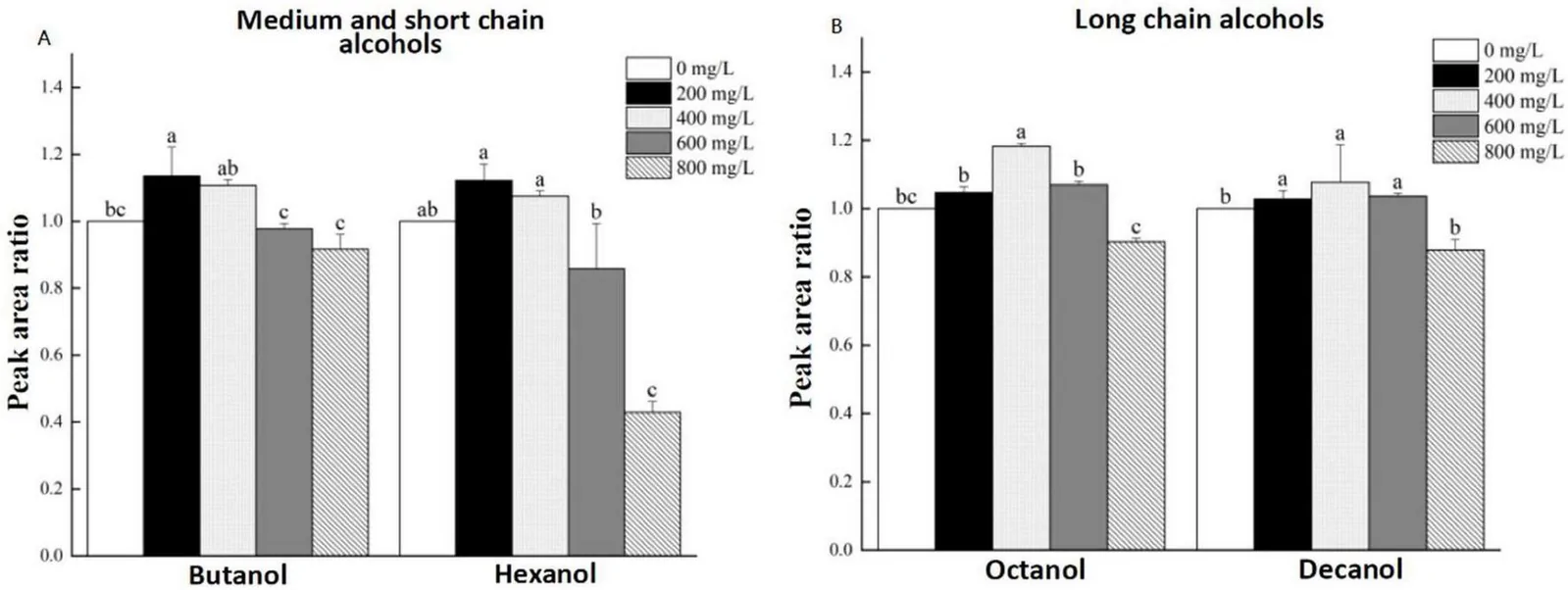
Effects of isoamyl alcohol on the volatility of aliphatic alcohols. **(A)** Medium- and short-chain alcohols. **(B)** Long-chain alcohols. Different lowercase letters denote significant differences via Duncan’s multiple range test (*p* < 0.05).

Isoamyl alcohol exerted typical bidirectional regulation on linear alcohols in binary models, showing promotion at low concentrations and inhibition at high concentrations (Figure 2). Short-chain alcohols were more sensitive to concentration changes than long-chain homologues, which was consistent with previous studies on alcoholic beverages (29). Concentrations below 400 mg/L promoted the headspace accumulation of these alcohols, while higher concentrations inhibited their volatilization. As shown in Table 2, the regulation effect of hexanol changed from a promotion rate of −12.14% to an inhibition rate of 57.08%, and octanol only fluctuated within a narrow range of −4.71 to 9.69%.

#### 3.1.3 Terpenoid derivatives

**FIGURE 3:**
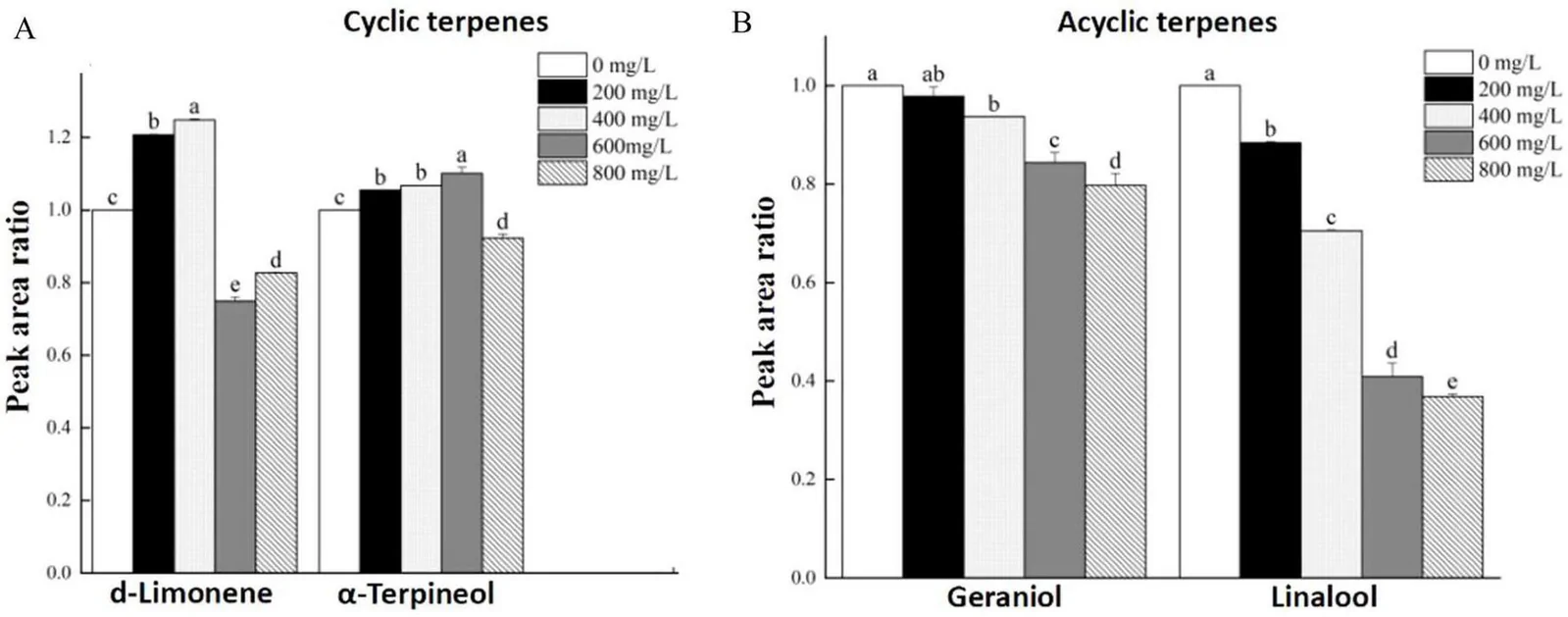
Effects of isoamyl alcohol on the volatility of terpenoids. **(A)** Acyclic Terpenols. **(B)** Monocyclic terpenes. Different lowercase letters denote significant differences via Duncan’s multiple range test (*p* < 0.05).

Isoamyl alcohol exerted entirely different regulatory effects on acyclic terpenols and monocyclic terpenes (Table 2). This distinct regulatory pattern is rarely observed in grain-type baijiu, which is mainly attributed to the low natural content of terpenoids in grain raw matrices. Acyclic geraniol and linalool exhibited continuous concentration-dependent volatility inhibition (*p* < 0.05). Linalool showed higher sensitivity, with a maximum inhibition rate of 63.14% at the isoamyl alcohol concentration of 800 mg/L. In contrast*, D*-limonene and α-terpineol exhibited promotion effects at low isoamyl alcohol concentrations and inhibitory effects at high concentrations, with their maximum promotion effects occurring at 400 and 600 mg/L, respectively. Under the same treatment conditions, *D*-limonene showed more significant promotion on volatile release (Figure 3).

#### 3.1.4 Minor characteristic odorants

**FIGURE 4:**
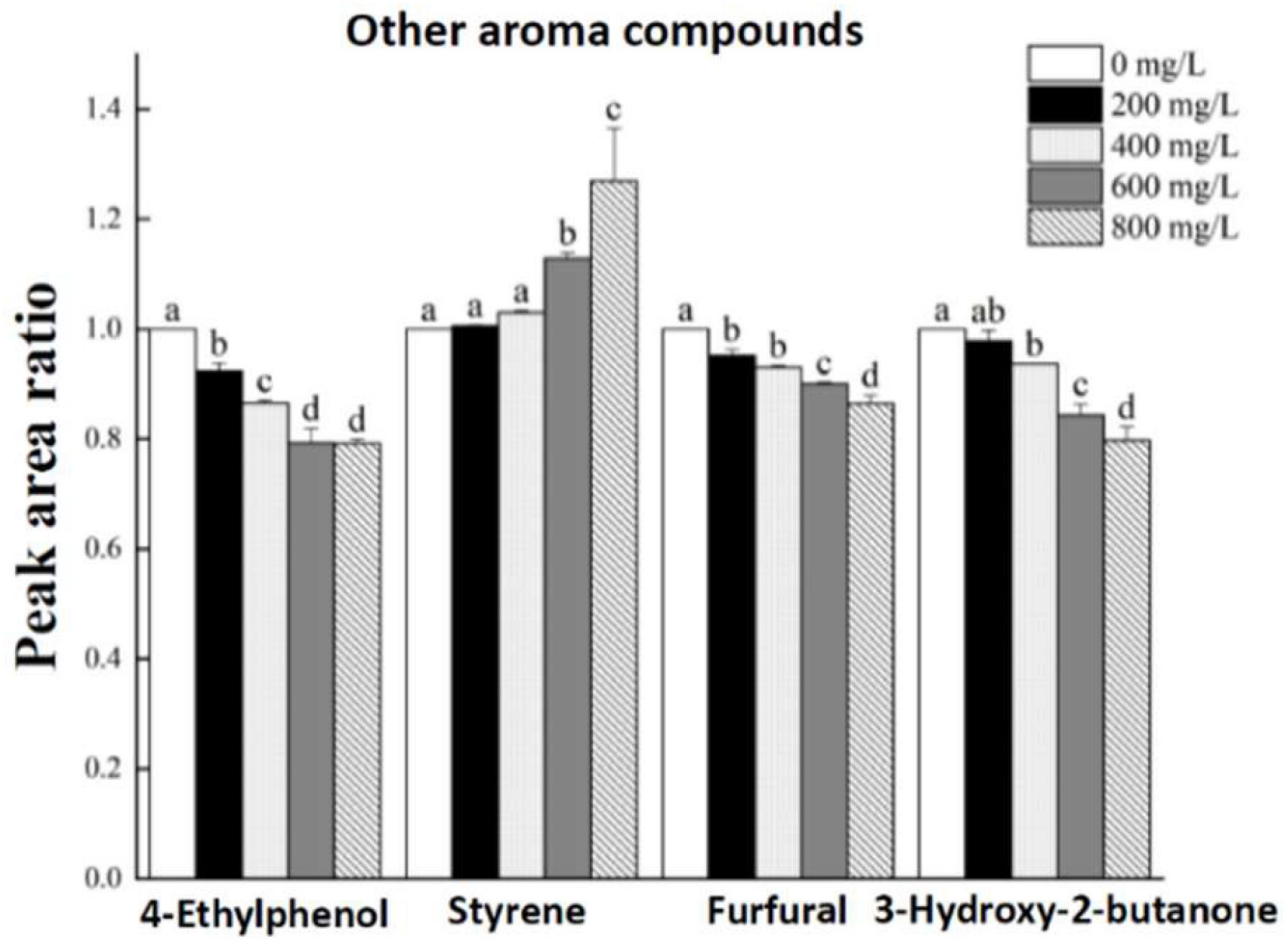
Effects of isoamyl alcohol on the volatility of minor characteristic typical odorants in navel orange brandy. Different lowercase letters denote significant differences via Duncan’s multiple range test (*p* < 0.05).

In binary model systems, isoamyl alcohol inhibited the volatilization of polar minor odorants, whereas it steadily promoted the release of weakly polar styrene (Table 2). The volatility of furfural, 4-ethylphenol and 3-hydroxy-2-butanone decreased gradually with the increase in isoamyl alcohol concentration, while the headspace abundance of styrene increased correspondingly (Figure 4).

### 3.2 Monomeric odorant olfactory threshold

To clarify odor interaction rules from the olfactory perception perspective, it is necessary to determine the olfactory thresholds of isoamyl alcohol and individual typical odorants in uniform binary hydroalcoholic model systems. Although olfactory threshold data of some compounds have been reported in previous studies (30–34), considerable discrepancies exist due to variations in ethanol concentration and matrix composition. Hence, the sigmoidal curve fitting method was applied to re-determine all monomeric olfactory thresholds in the 54% (v/v) ethanol simulated system.

**FIGURE 5:**
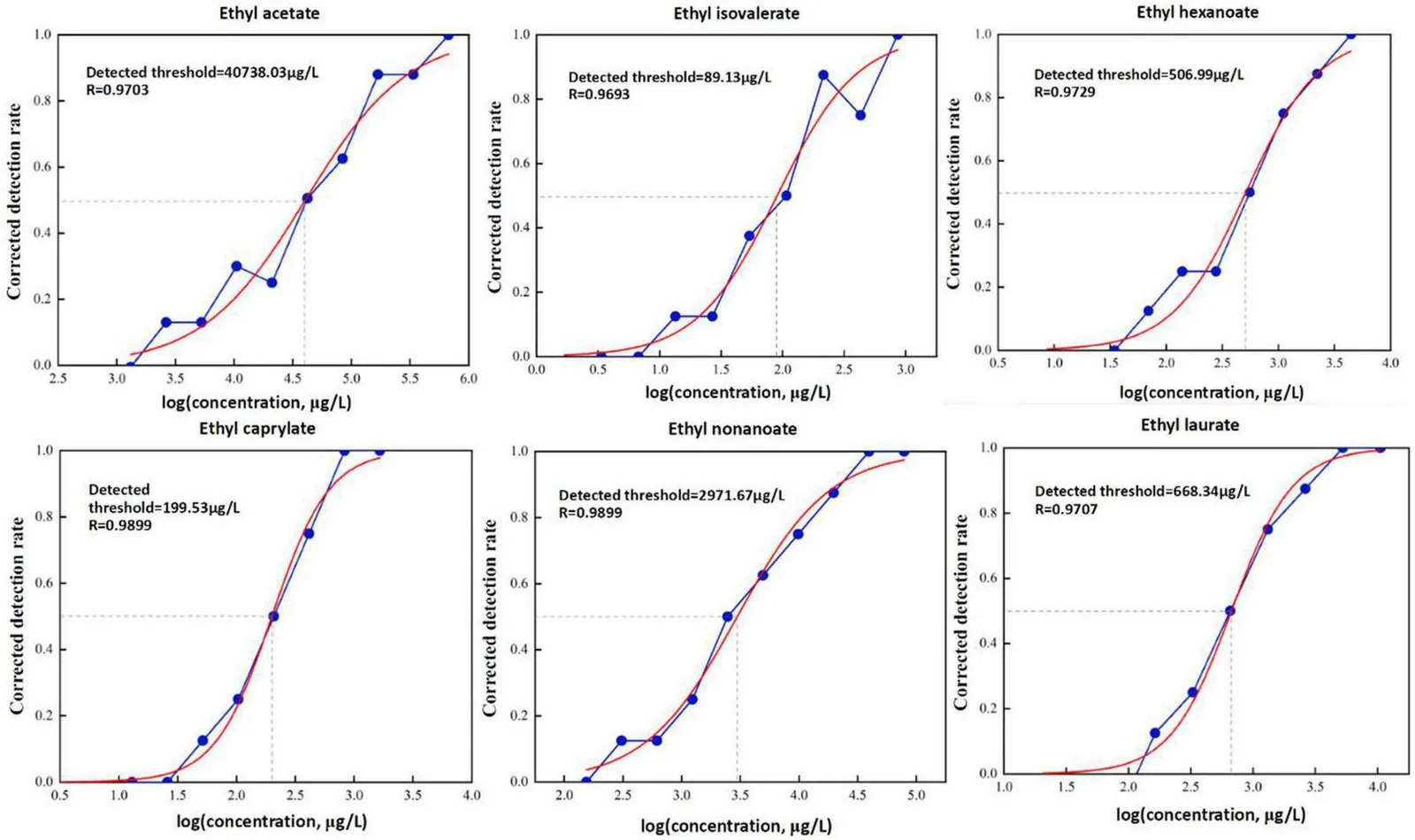
Sigmoidal olfactory threshold curves for six key fatty acid ethyl esters.

**FIGURE 6:**
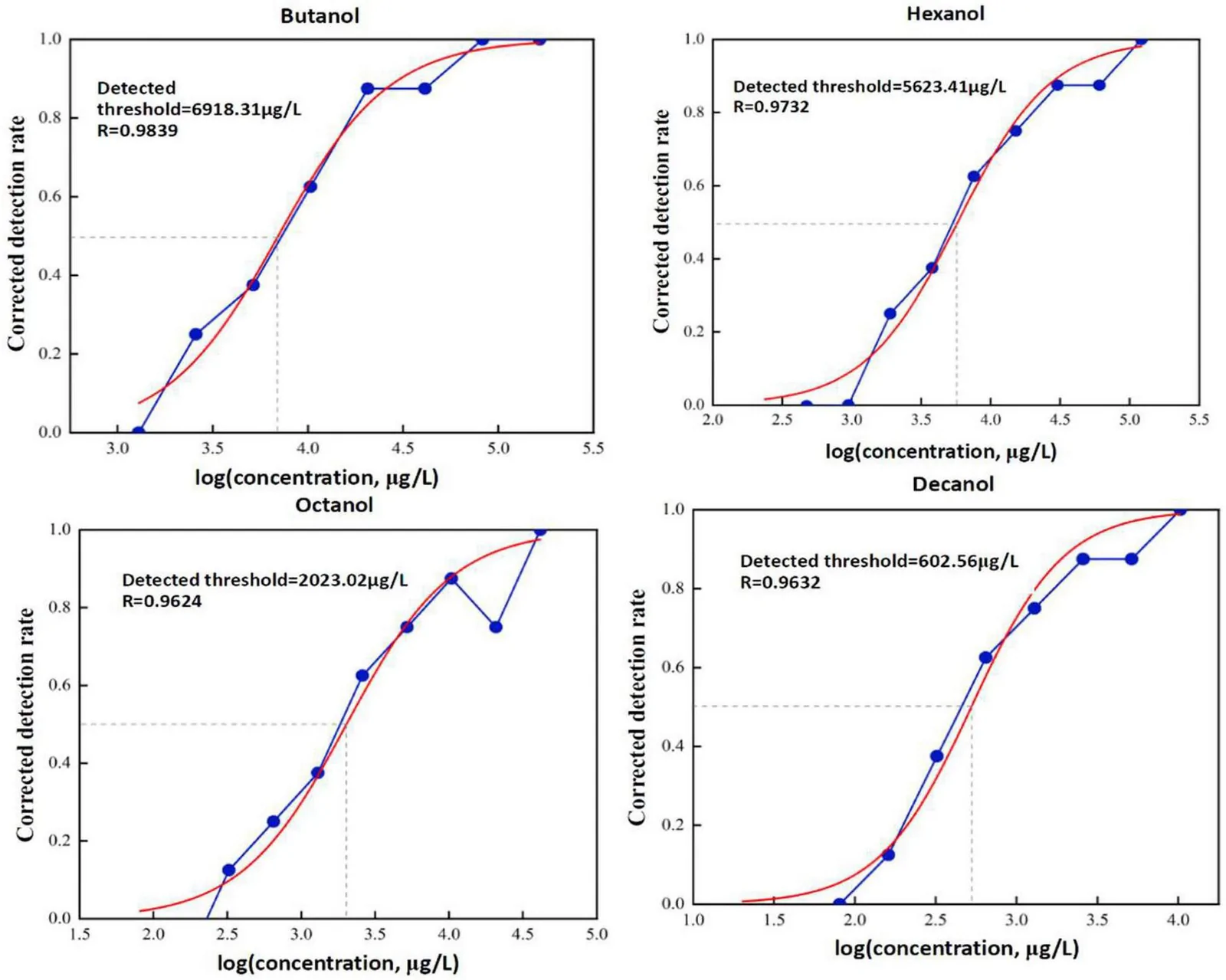
Sigmoidal olfactory threshold curves for four linear aliphatic alcohols.

**FIGURE 7:**
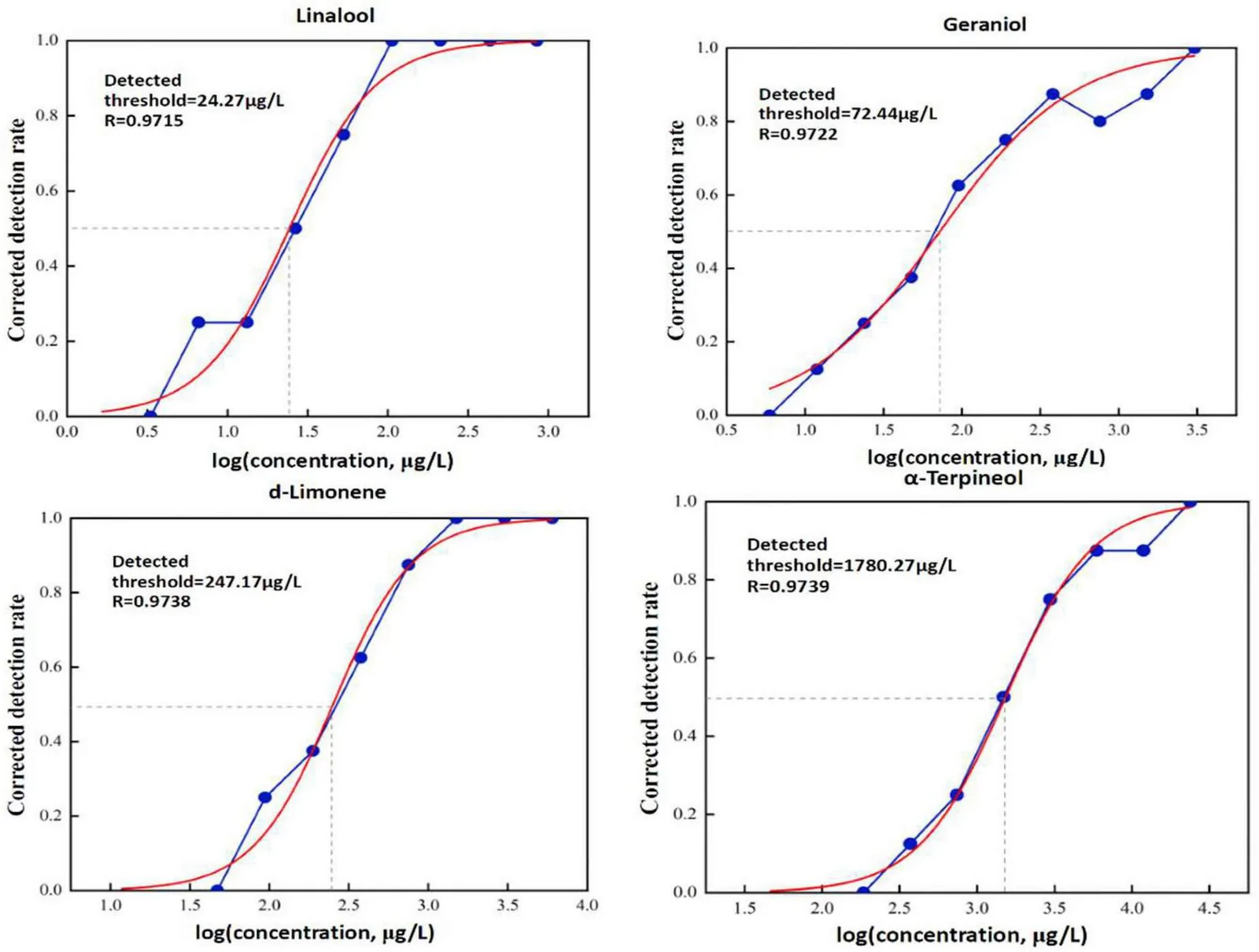
Sigmoidal olfactory threshold curves for four major terpenoid derivatives.

**FIGURE 8:**
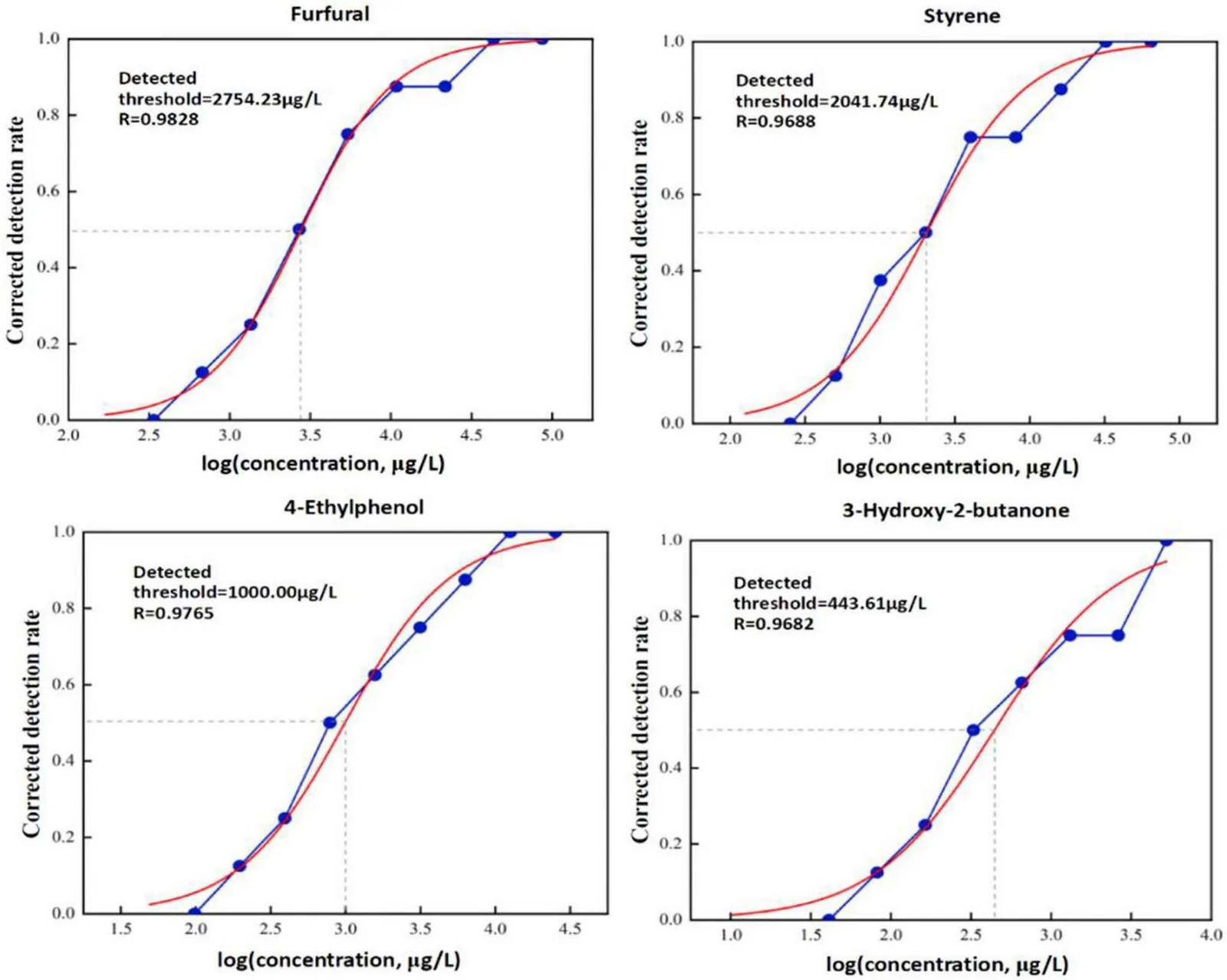
Sigmoidal olfactory threshold curves for minor characteristic typical odorants.

Table 3 lists the olfactory thresholds obtained via sigmoidal curve fitting, with all coefficients of determination *R*^2^ above 0.96 (Figures 5–8). Most thresholds measured in this study were higher than the published values determined in 46% (v/v) ethanol solution, whereas decanol and furfural exhibited relatively lower thresholds. This indicates that ethanol concentration is a vital factor affecting olfactory detection thresholds. Medium-chain ethyl esters exhibited the most prominent threshold differences between the two matrix systems. In terms of linear aliphatic alcohols, their olfactory thresholds decreased gradually with the extension of carbon chain length, which was consistent with the results of previous sensory studies on alcoholic beverages (35, 36). Apart from ethanol concentration, these threshold discrepancies may also be attributed to sensory panel constitution, experimental testing routines, ambient temperature, chemical purity of target compounds and sample matrix constitution (37).

**TABLE 3:** Sigmoidal curve-fitted olfactory thresholds of single typical odorants.

No.	Compound	Odor description	Threshold (μg/L)	
			Literature threshold^a^	Measured threshold^b^
1	Isoamyl alcohol	Whiskey, malt, burnt aroma	70,000	79799.49
2	Ethyl acetate	Pineapple aroma	32,600	40738.03
3	Ethyl isovalerate	Apple aroma, fruity aroma	6.89	89.13
4	Ethyl hexanoate	Fruity aroma, sweet aroma	55.3	506.99
5	Ethyl octanoate	Fruity aroma, floral aroma, pineapple aroma	12.9	199.53
6	Ethyl nonanoate	Fruity aroma	1,200	2971.67
7	Ethyl laurate	Waxy aroma, fruity aroma	500	668.34
8	Butanol	Fruity aroma	2,730	6918.31
9	Hexanol	Floral aroma	5,370	5623.41
10	Octanol	Fruity aroma	1,100	2023.02
11	Decanol	Sweet aroma, floral aroma	770	602.56
12	Geraniol	Rose aroma, geranium aroma	30	72.44
13	Linalool	Floral aroma, lavender aroma	15	24.27
14	*D*-Limonene	Orange aroma, lemon aroma	13.5	247.17
15	α-Terpineol	Fatty aroma, anise aroma, mint aroma	–	1780.27
16	4-Ethylphenol	Smoky aroma	600	1000.00
17	Furfural	Sweet aroma, almond aroma	3,000	2754.23
18	Styrene	Plastic odor, pungent odor	1,400	2041.74
19	3-Hydroxy-2-butanone	Butter aroma, creamy aroma	259	443.61

*^a^*Literature thresholds were determined in 46% (v/v) ethanol-water matrix.

*^b^*Measured thresholds were obtained in the 54% (v/v) binary hydroalcoholic model of this study; “–” represents unavailable published data.

Figures 5–8 separately present the sigmoidal fitting curves for esters, linear alcohols, terpenoids and minor characteristic odorants. All fitting models exhibited high correlation coefficients, which verified that the combined 3-AFC and sigmoidal curve method could realize accurate quantification of olfactory thresholds.

### 3.3 Olfactory interplay of binary isoamyl alcohol–typical odorant mixtures

Combined with the gas-liquid distribution results in section 3.1 and monomeric olfactory threshold data in section 3.2, the olfactory interaction characteristics of binary odorant mixtures were further categorized. Three interaction models, including masking, additive, synergistic effects, were identified among 18 binary blends constructed in hydroalcoholic model systems (Table 4).

**TABLE 4:** Olfactory interaction classification of binary isoamyl alcohol-odorant mixtures.

No.	Binary mixture combination	Measured threshold (μg/L)	Theoretical threshold (μg/L)	Threshold ratio	Interaction type
1	Isoamyl alcohol + Ethyl acetate	63973.48	18365.38	3.483	Masking effect
2	Isoamyl alcohol + Ethyl hexanoate	84139.51	43651.58	1.928	Masking effect
3	Isoamyl alcohol + Ethyl isovalerate	77983.01	49856.57	1.564	Masking effect
4	Isoamyl alcohol + Ethyl octanoate	54701.60	50118.72	1.091	Masking effect
5	Isoamyl alcohol + Ethyl nonanoate	26001.60	29716.66	0.875	Additive effect
6	Isoamyl alcohol + Ethyl laurate	76736.15	36307.81	2.113	Masking effect
7	Isoamyl alcohol + Butanol	10115.80	31988.95	0.316	Synergistic effect
8	Isoamyl alcohol + Hexanol	10592.53	31550.05	0.336	Synergistic effect
9	Isoamyl alcohol + Octanol	23227.38	28707.81	0.809	Additive effect
10	Isoamyl alcohol + Decanol	16982.44	30549.21	0.556	Additive effect
11	Isoamyl alcohol + Linalool	15170.50	21827.30	0.695	Additive effect
12	Isoamyl alcohol + Geraniol	37153.52	35481.34	1.047	Masking effect
13	Isoamyl alcohol + *D*-Limonene	5223.96	27989.81	0.187	Synergistic effect
14	Isoamyl alcohol + α-Terpineol	27039.58	36475.39	0.741	Additive effect
15	Isoamyl alcohol + Furfural	17988.71	23988.33	0.750	Additive effect
16	Isoamyl alcohol + 4-Ethylphenol	53703.18	26302.68	2.042	Masking effect
17	Isoamyl alcohol + Styrene	8184.65	20323.57	0.403	Synergistic effect
18	Isoamyl alcohol + 3-Hydroxy-2-butanone	33884.42	36897.76	0.918	Additive effect

Masking interactions mainly existed in ester-containing mixtures, while additive and synergistic effects were predominant in alcohol and terpene combinations. The distribution rule of masking effects was consistent with the volatility inhibition trends determined by HS-SPME-GC-MS, and synergistic interactions could enhance the overall sensory intensity of binary mixed systems. It should be noted that the above interaction patterns were only verified in pairwise binary mixtures, and complex multi-component odor coupling effects in actual citrus brandy need to be further explored.

#### 3.3.1 Binary mixtures of isoamyl alcohol and fatty acid ethyl esters

**FIGURE 9:**
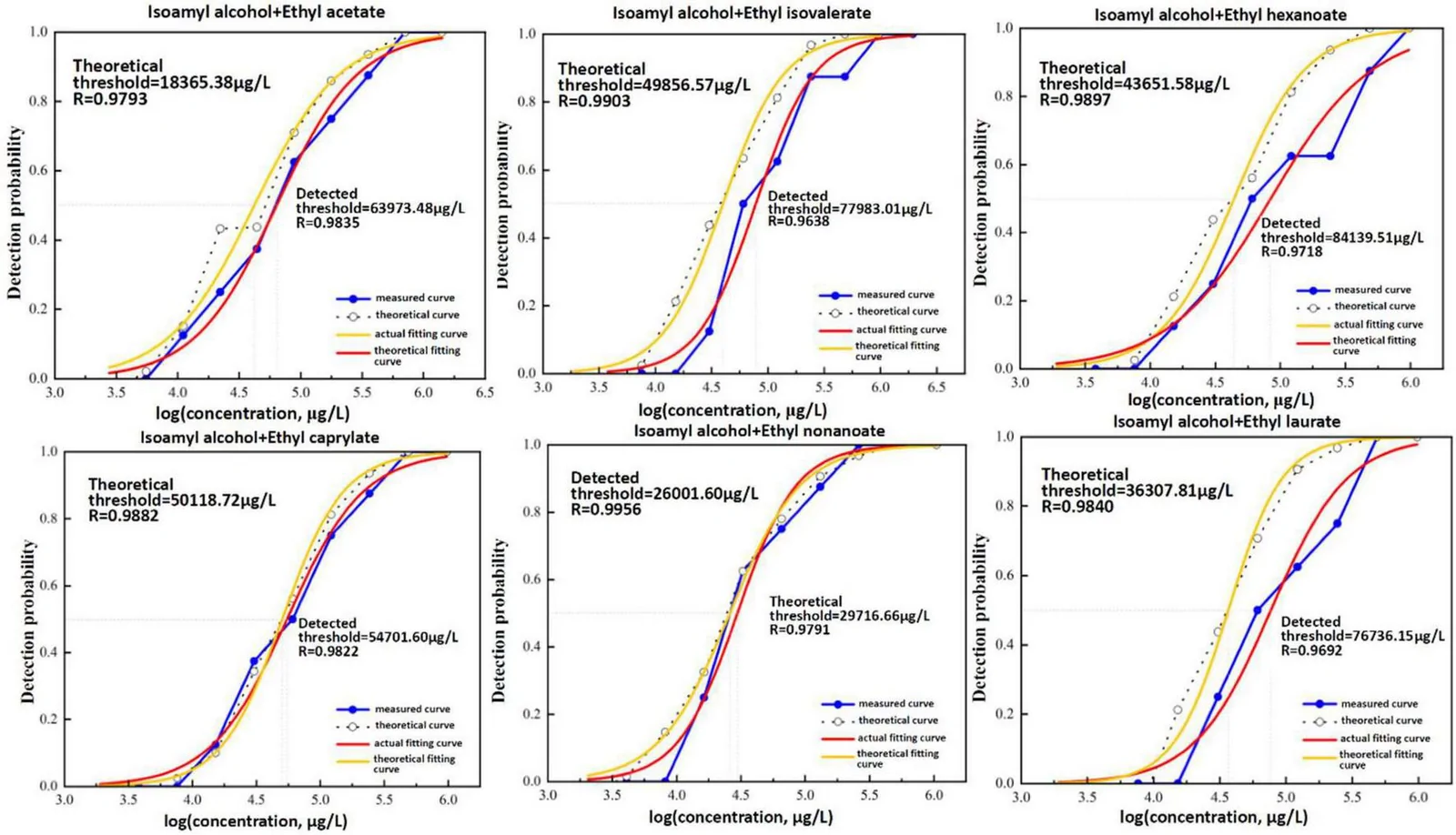
Sigmoidal threshold modeling for binary mixtures of isoamyl alcohol and fatty acid ethyl esters.

Sigmoidal fitting curves (Figure 9) showed that short-chain ethyl esters exerted masking effects when mixed with isoamyl alcohol, while ethyl nonanoate exhibited additive interactions. The masking effect gradually weakened with the extension of ester carbon chain length, which was consistent with the volatility inhibition trends determined by GC-MS analysis.

#### 3.3.2 Binary mixtures of isoamyl alcohol and linear aliphatic alcohols

**FIGURE 10:**
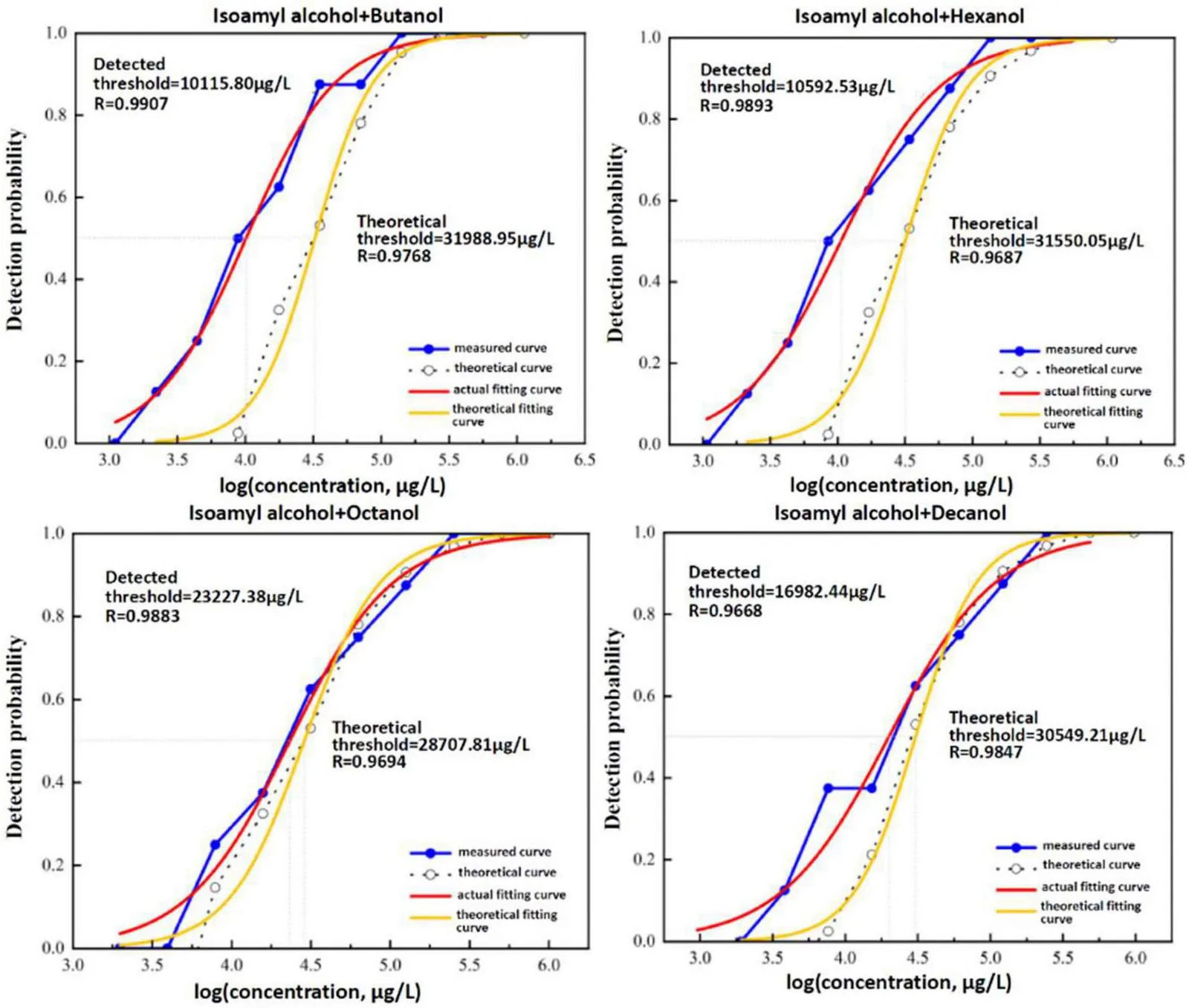
Sigmoidal threshold modeling for binary mixtures of isoamyl alcohol and aliphatic alcohols.

As illustrated in Figure 10, short- and medium-chain linear alcohols including butanol and hexanol exerted distinct synergistic effects when blended with isoamyl alcohol, which effectively increased the overall olfactory intensity of mixed systems. In comparison, long-chain linear alcohols such as octanol and decanol only exhibited additive interactions, showing no significant enhancement in aroma perception.

#### 3.3.3 Binary mixtures of isoamyl alcohol and terpenoid derivatives

**FIGURE 11:**
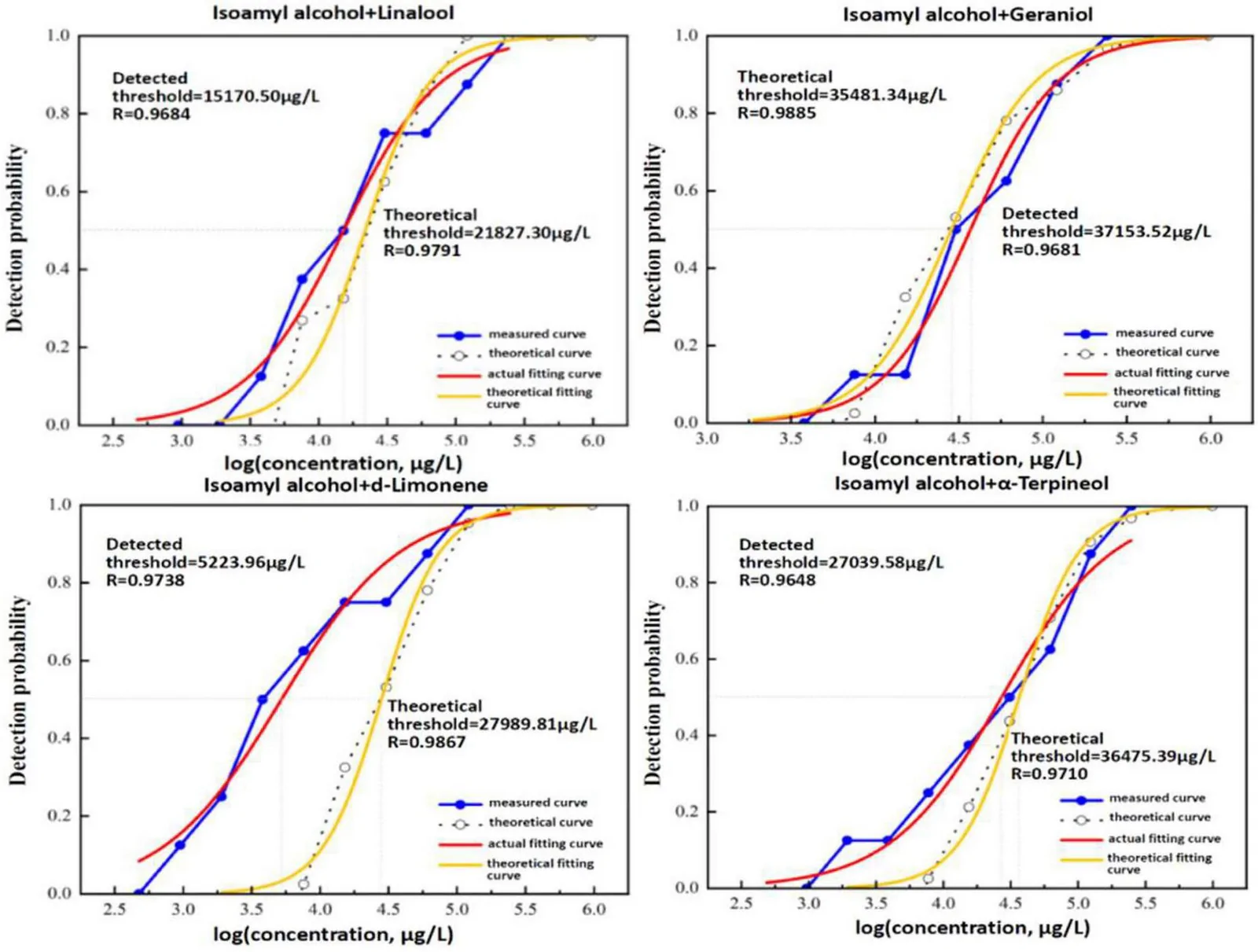
Sigmoidal threshold modeling for binary mixtures of isoamyl alcohol and terpenoid derivatives.

As shown in Figure 11, geraniol exerted masking effects when blended with isoamyl alcohol, whereas linalool and α-terpineol exhibited additive interactions. In contrast, *D*-limonene produced remarkable synergistic effects to strengthen the overall aroma intensity of the mixture.

#### 3.3.4 Binary mixtures of isoamyl alcohol and minor odorants

**FIGURE 12:**
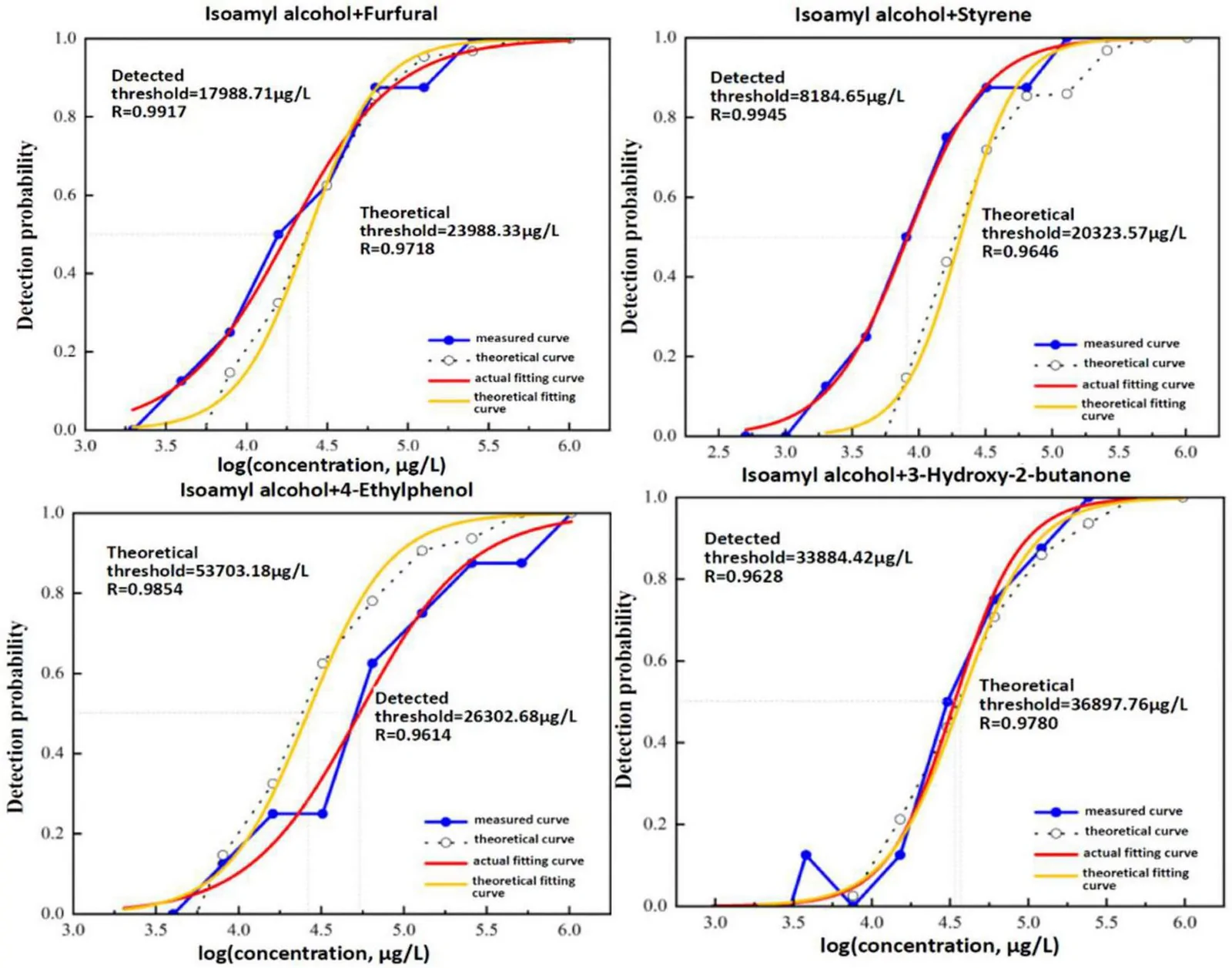
Sigmoidal threshold modeling for binary mixtures of isoamyl alcohol and minor characteristic typical odorants.

As illustrated in Figure 12, different minor compounds displayed varied olfactory interaction behaviors with isoamyl alcohol. Furfural and 3-hydroxy-2-butanone exhibited additive interactions, styrene exerted synergistic effects, and 4-ethylphenol exerted evident masking effects in their respective binary systems.

## 4 Discussion

### 4.1 Analysis of gas-liquid distribution and volatile release characteristics

Headspace analysis confirmed that diverse aroma compounds differed markedly in gas-liquid distribution behavior and volatile performance in simulated navel orange brandy matrix. These differences occurred not only across different classes of aroma compounds, but also among homologues with different carbon chain lengths after the addition of isoamyl alcohol.

Such distinctions in volatile behavior might be affected by two core influencing factors. It is speculated that the inherent physicochemical characteristics of each compound define its fundamental volatile tendency. Meanwhile, it is hypothesized that intermolecular association and steric hindrance between isoamyl alcohol and other aroma molecules could serve as important regulatory factors (38, 39). It is presumed that disparities in molecular structure and functional groups may result in varying degrees of intermolecular binding. In speculative terms, short-chain compounds are likely to possess weaker intermolecular forces and tend to volatilize more easily into the gas phase, while long-chain homologues might show stronger intermolecular binding and remain in the liquid phase. This rule and speculative view are in good agreement with previous literature reports (40).

These findings could partly illustrate the sensory differences of aroma compounds at similar concentrations, and lay a foundation for subsequent research on olfactory thresholds and aroma interactions. They also provide practical guidance for actual flavor regulation. As this study was carried out in simplified model systems, further verification using authentic wine samples is necessary.

### 4.2 Characterization of olfactory thresholds of individual aroma components

The olfactory thresholds determined in this study deviated from those documented in previous literature, revealing that ethanol content might act as a vital factor regulating olfactory detection thresholds. To simulate the actual characteristics of navel orange distilled spirits, the alcoholic strength adopted in this experiment was higher than that applied in most published studies. It is considered that elevated ethanol concentration may exert an evident olfactory masking effect on nasal perception, which could raise the minimum detectable concentration and further increase the olfactory thresholds of aroma compounds (41, 42). Apart from ethanol-induced sensory suppression, differences in wine matrix conditions and presumed interactive effects among coexisting flavor constituents also account for such numerical differences (43). In this regard, the threshold data obtained in the present study are more representative of the real sensory environment of high-alcohol navel orange distilled spirits.

Besides the effect of ethanol concentration, such numerical differences are also associated with various experimental variables. These confounding factors, including sensory panel composition, evaluation protocols, testing temperature, sample purity and sample matrix conditions, can all affect olfactory detection thresholds (37).

Distinct differences in olfactory perception performance exist among different types of aroma substances, and gradual variations can also be found within the same chemical category. It is speculated that these differences may be related to basic molecular structures, and are more likely affected by their intrinsic odor activity, molecular polarity and existing form in alcoholic systems (44). These inherent properties might collectively lead to varied sensory sensitivity during olfactory identification, and the acquired threshold data can also provide reliable theoretical support for targeted flavor optimization and scientific aroma blending of navel orange brandy.

### 4.3 Elucidation of olfactory interaction patterns in binary mixtures

Binary mixtures consisting of isoamyl alcohol and other aroma compounds presented varied olfactory interaction behaviors in this study. It is hypothesized that the formation of masking, synergistic and additive effects may be mainly ascribed to competitive binding between odor molecules and olfactory receptors, as well as the superposition or counteraction of sensory attributes and distinct aroma release features within mixed systems (45).

It is considered that differences in molecular structure, polarity and sensory properties might be important factors affecting the interactive relationship between isoamyl alcohol and various flavor substances (46). Low-molecular-weight esters generally possess relatively strong polarity and sharp sensory traits, which may easily cause perceptual inhibition when mixed with isoamyl alcohol. As carbon chain length and molecular weight increase, the gradual decline in molecular polarity and milder odor perception are presumed to alleviate such inhibitory influence, which may further facilitate the transition toward additive interaction. In comparison, short-chain alcohols have relatively similar structural and olfactory features to isoamyl alcohol, which is likely to bring about sensory complementation and induce synergistic effects. The growing molecular volume and steric hindrance may weaken such complementary action, and the interaction tends to develop into a simple additive state. This changing trend conforms to the inherent volatile properties of related aroma substances, which suggests that physicochemical properties determined by molecular structures may play a vital role in modulating olfactory interaction modes (47).

Notably, theoretical thresholds calculated via the Feller additive formula are strictly based on the hypothesis of independent olfactory detection events, ignoring actual molecular interactions, olfactory receptor competitive binding and sensory neural cross-regulation among different odorants, which endows this method with inherent application limitations.

Several blending combinations exhibited typical extreme interaction effects including strong masking and prominent synergism. The present findings provide reliable theoretical references for reasonable aroma formulation and targeted flavor regulation of navel orange distilled spirits.

## 5 Conclusion

This study established 54% (v/v) binary hydroalcoholic models to simulate the actual matrix of navel orange brandy, and investigated the effects of isoamyl alcohol on volatilization characteristics and olfactory interactions of typical aroma compounds. Isoamyl alcohol exhibited concentration-dependent regulatory effects on aroma release. It suppressed the volatilization of fatty acid ethyl esters with inhibitory intensity varying with carbon chain length, and exerted bidirectional actions on aliphatic alcohols. Terpenoids and trace aroma compounds also showed differentiated volatile responses. Sensory evaluation identified three olfactory interaction modes including masking, additive and synergistic effects, which were highly consistent with instrumental analysis results. These diverse phenomena may result from differences in molecular physicochemical properties and intrinsic olfactory features of odorants, and are presumed to be linked to intermolecular hydrogen bonding and hydrophobic aggregation in hydroalcoholic systems. It should be clarified that all mechanistic inferences are summarized on the basis of macroscopic results, and no direct microscopic evidence has been obtained to confirm these interactions.

The obtained results can provide a reliable theoretical basis for isoamyl alcohol regulation and targeted flavor modulation in navel orange brandy production. This research was carried out in simplified binary systems, which excluded complex endogenous components and multi-component flavor interactions in real products, and lacked verification in practical production scenarios. Subsequent studies will establish multi-component simulation systems, verify the above inferred interaction mechanisms via microscopic techniques, and carry out field verification to optimize the overall sensory quality of citrus distilled spirits.

## Data Availability

The original contributions presented in the study are included in the article/supplementary material, further inquiries can be directed to the corresponding authors.
